# Comparison of different implant-based breast reconstruction strategies: A systematic review and Bayesian network meta-analysis

**DOI:** 10.1097/JS9.0000000000005090

**Published:** 2026-03-17

**Authors:** Junlei Li, Bin Pan, Tengjiang Long, Zeyu Yang, Yingfan Chen, Ziying Yi, Yao Li, Yiceng Sun, Tingjie Yin, Xi Chen, Yinde Huang, Supeng Yin, Fan Zhang

**Affiliations:** a Graduate School of Medicine, Chongqing Medical University, Chongqing, China; b Department of Breast and Thyroid Surgery, Chongqing General Hospital, Chongqing University, Chongqing, China

**Keywords:** breast reconstruction, delayed, immediate, network meta-analysis, prepectoral, subpectoral

## Abstract

**Background:**

Implant-based breast reconstruction is the most common postmastectomy approach, but the optimal combination of implant plane (prepectoral vs. subpectoral) and timing (immediate vs. delayed) remains controversial.

**Methods:**

We systematically searched five databases through March 2025 and included observational studies that compared immediate subpectoral breast reconstruction (ISBR), delayed subpectoral breast reconstruction, immediate prepectoral breast reconstruction (IPBR), and delayed prepectoral breast reconstruction (DPBR). We performed pairwise meta-analyses and Bayesian network meta-analyses (NMA) using R (version 4.4.1). To enhance interpretability, the surface under the cumulative ranking curve (SUCRA) was used to assess the probability that each strategy was the optimal choice for each prespecified outcome.

**Results:**

Thirty-one retrospective studies were included. In the NMA, no statistically significant differences were found among the strategies for overall complications, hematoma, or necrosis. Compared with ISBR, IPBR was associated with lower risks of reoperation and wound dehiscence. ISBR was associated with the greatest risk of implant loss. In terms of volume, direct evidence favored DPBR over IPBR, and the SUCRA suggested that delayed strategies are advantageous. No statistically significant differences were observed across the BREAST-Q “satisfaction with breasts,” “sexual well-being” scales, and pain.

**Conclusions:**

Across strategies, overall safety is broadly comparable. Prepectoral reconstruction tends to reduce the reoperation rate and capsular contracture rate, whereas subpectoral approaches have fewer constraints regarding flap quality. Delayed reconstruction better supports volume optimization and flexibility, whereas immediate approaches have lower follow-up demands and costs. Surgical planning should integrate flap quality, recovery burden, and patient goals for more individualized treatment selection.

## Introduction

Breast cancer is the most prevalent malignancy among women worldwide^[^1^]^. As mastectomy is a fundamental surgical prevention and treatment strategy for breast cancer, many patients inevitably undergo this procedure^[^2,3^]^. However, with increasing public demand for esthetics, the loss of a breast is unacceptable for most women^[^4^]^. Therefore, breast reconstruction (BR) after mastectomy plays a crucial role in the management of patients with breast cancer^[^5,6^]^.

HIGHLIGHTS

- First network meta-analysis comparing four reconstruction strategies for safety and patient outcomes.
- Overall complication rates were broadly similar across the four reconstruction strategies.
- Immediate prepectoral reconstruction reduced reoperation and wound dehiscence versus immediate subpectoral.
- Immediate subpectoral reconstruction showed the highest risk of implant loss among the four strategies.
- Delayed reconstruction supported larger implant volumes without compromising overall safety.

Compared with autologous BR, implant-based BR is the most common form of BR and has several advantages, such as simpler surgical procedures and shorter recovery times^[^7,8^]^. However, the optimal plane for implant placement (prepectoral vs. subpectoral) and the timing of reconstruction (immediate vs. delayed) remain controversial in clinical practice^[^9^]^. Since the introduction of silicone implants in the 1960s, attempts at prepectoral BR have been limited by high complication rates and suboptimal esthetic outcomes. In contrast, in subpectoral BR, the implant is placed under the pectoralis major muscle. This procedure has been widely adopted because it reduces implant visibility and palpability and decreases the risk of capsular contracture^[^10^]^. However, the dissection of the pectoralis major in subpectoral reconstruction may result in animation deformities, postoperative pain, and restricted shoulder mobility^[^11^]^. Advances in surgical techniques and the use of acellular dermal matrix and other biological meshes have lessened many of the historical drawbacks of prepectoral BR, such as visible implant contours and capsular contracture^[^12^]^. Prepectoral BR preserves the integrity of the pectoralis major muscle, which may decrease postoperative morbidity and maintain a more natural breast shape and dynamics^[^13^]^. Nevertheless, concerns persist regarding an increased risk of skin flap necrosis, rippling, and implant exposure, especially in patients with limited soft tissue coverage^[^14^]^.

The choice of timing is equally critical. Tissue expander (TE)-based delayed BR offers flexibility, but it requires repeated expansions and a second surgery. This process often results in additional anesthesia risk, a longer treatment duration, and extended psychosocial stress^[^15^]^. In contrast, immediate BR avoids repeated expansions, which may improve patient comfort, increase patient satisfaction, and provide early psychological benefits^[^13^]^. Nonetheless, immediate BR depends on the quality of the skin flap and the available soft tissue. This constraint limits the maximum volume of the implant and complicates postoperative management, thereby impacting patient satisfaction^[^16^]^. Therefore, the selection of immediate versus delayed BR requires a balance of flexibility against soft tissue constraints and recovery burden.

Although extensive research on BR has been conducted, previous meta-analyses were limited to pairwise comparisons that focused on implant plane and timing. No study has systematically evaluated all four surgical methods within a unified analytical framework. In this study, a network meta-analysis was conducted to comprehensively assess surgical safety and patient satisfaction across four BR techniques: immediate subpectoral breast reconstruction (ISBR), delayed subpectoral breast reconstruction (DSBR), immediate prepectoral breast reconstruction (IPBR), and delayed prepectoral breast reconstruction (DPBR). Notably, we ranked all surgical methods with respect to different indices to elucidate their respective clinical advantages and limitations and to provide an evidence-based medical basis for the selection of BR. The study followed the TITAN Guidelines 2025^[^17^]^.

## Methods

### Design and registration

This network meta-analysis reports the different indices of four surgical methods in accordance with the Preferred Reporting Items for Systematic Reviews and Meta-Analyses (PRISMA) and Assessing the Methodological Quality of Systematic Reviews (AMSTAR) guidelines^[^18,19^]^. This study is registered in the International Prospective Register of Systematic Reviews (PROSPERO).

### Search strategy

We performed a systematic literature search of the PubMed, Medline, Cochrane Library, Web of Science, and EMBASE databases up to March 2025 to identify all relevant studies. The keywords used were (“prepectoral” OR “suprapectoral” OR “subcutaneous” OR “premuscular” OR “supramuscular” OR “subpectoral” OR “submuscular” OR “retropectoral”) AND (“immediate reconstruction” OR “delayed reconstruction”) AND (“implant” OR “tissue expander”) AND (“breast reconstruction” OR “mammaplasty”). In addition, the reference lists of the retrieved articles were reviewed to obtain other eligible studies.

Two reviewers independently conducted the literature search, and all disagreements were resolved by consensus. The abstracts of the retrieved studies were reviewed, and studies were excluded if they were deemed irrelevant. The full texts of the remaining studies were reviewed to determine their eligibility. Discrepancies were resolved through discussion with a third reviewer.

### Inclusion and exclusion criteria

Studies were considered eligible if they met the following inclusion criteria: (1) study design: observational study; (2) population: patients who had undergone implant-based BR; (3) intervention: studies comparing at least two surgical methods, including IPBR, ISBR, DPBR, and DSBR; (4) outcomes: studies reporting at least two outcomes of interest mentioned below; (5) language: studies that were published in English; and (6) time window: articles published within 10 years prior to the final search date (2015–2025). The exclusion criteria were as follows: (1) conference reports, abstracts, reviews, case reports, animal studies, commentaries, discussions, letters, and noncontrol studies; (2) duplicate articles; and (3) publications outside the 10-year window (before 2015).

### Data extraction

Two investigators independently extracted the following data from each study: (1) characteristics of the study, including the first author, year and country of publication, study design, and surgical methods; (2) surgical safety, including the overall complication rate, infection rate, hematoma rate, seroma rate, necrosis rate, and implant loss rate (meaning that the implant was removed and not replaced or was removed and replaced because of a complication); and (3) patient-reported outcomes, including implant volume, breast satisfaction, sexual well-being, pain, psychosocial well-being, and physical well-being.

### Quality assessment

Randomized controlled trials (RCTs) and nonrandomized studies (NRSs) were classified by their methodological design. NRSs included prospective cohort and case‒control studies. The quality assessment of the included studies was independently conducted by two investigators using the Cochrane Collaboration tool for RCTs and the Newcastle‒Ottawa Scale (NOS) for NRSs, in accordance with the Cochrane Collaboration guidelines^[^20^]^. Differences were resolved by a third investigator or through team consensus.

### Statistical analysis

Pairwise meta-analyses (PMAs) were conducted when at least two studies reported the same direct comparison. All analyses were performed in R (version 4.4.1) using standard packages for pairwise and network meta-analyses (NMA). Continuous data were summarized using the mean difference (MD) with 95% confidence interval (CI), and dichotomous outcomes were analyzed using the odds ratio (OR) with corresponding 95% CI. Heterogeneity was assessed using the *Q* test, and the *I*^2^ statistic (*P* < 0.05 for *Q* or *I*^2^ > 50%) served as an indicator of substantial heterogeneity. A random-effects model was used when heterogeneity was substantial; otherwise, a fixed-effects model was used.

For the Bayesian network meta-analysis, JAGS (version 4.3.0) served as the Markov chain Monte Carlo engine. The resulting effects are reported as the posterior median (MD) or OR with corresponding 95% CI. The analysis was performed using 1,000 burn-ins, 50 000 iterations, and 20 000 adaptation steps. Model fit and influence were examined using leverage plots. With respect to model selection, the choice between fixed-effects and random-effects models was determined by comparing the deviance information criterion (DIC) values to account for potential heterogeneity, with the model that yielded the lower DIC being selected as the preferred fit^[^21^]^. Global consistency was assessed by comparing the consistency and inconsistency models using the DIC. Loop-specific inconsistency tests were not performed because no comparisons in the observed network included both direct and indirect evidence.

For each outcome, we summarized treatment hierarchies using the surface under the cumulative ranking curve (SUCRA) and mean ranks. A higher SUCRA value indicates a higher probability that the treatment is the better method. Sensitivity analysis was conducted by sequentially excluding each included study to evaluate whether meaningful changes in the combined effect occurred. Publication bias and reporting bias were examined using comparison-adjusted funnel plots and Egger’s test.

## Results

### Literature search

**Figure 1.**
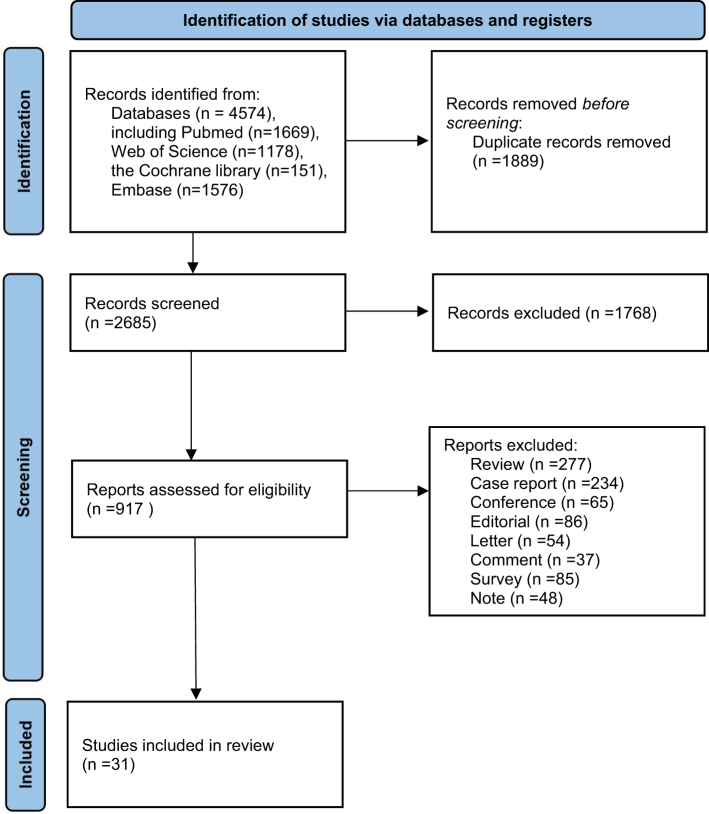
A PRISMA flowchart for the search and selection of eligible studies included in the network meta-analysis.

Employing the described search strategy, 4,574 potentially relevant articles were identified. After initial screening and the removal of duplicates, a full-text review was conducted to exclude articles that did not meet the inclusion criteria, resulting in the identification of 31 studies^[^22–52^]^. The PRISMA flow diagram shows the details of the article selection and exclusion procedure (Fig. 1).

### Study characteristics

The basic characteristics of the included studies are presented in Table 1. The mean age of the patients ranged from 34.2 to 56.2 years. All 31 publications were retrospective studies. The meta-analysis included four surgical methods: DPBR, IPBR, DSBR, and ISBR. All the studies were performed from 2015 to 2025. The overall quality of the 31 studies was assessed by the NOS score; all the studies were regarded as high quality, as indicated by a score of 7 or higher (Supplemental Digital Content Table S1, available at: http://links.lww.com/JS9/H88). The network relationships among surgical methods for each outcome are shown in Supplemental Digital Content Figure S1, available at: http://links.lww.com/JS9/H87. The random-effects and fixed-effects model fit for the NMA is shown in leverage plots (Supplemental Digital Content Figure S2, available at: http://links.lww.com/JS9/H87). No significant inconsistencies were detected across the outcomes of different treatment strategies in the NMA (Supplemental Digital Content Figure S3, available at: http://links.lww.com/JS9/H87). Publication bias in the included studies was assessed using calibration funnel plots and Egger’s test. No significant evidence of publication bias was found (Supplemental Digital Content Figure S4, available at: http://links.lww.com/JS9/H87).

**Table 1:** Characteristics of the 31 included studies.

Study	Year	Method	Age	BMI (kg/m^2^)	Sample size (*n*)
Zhu *et al*	2016	DPBR	50.48 ± 8.83	27.77 ± 5.35	50
		DSBR	52.69 ± 12.18	27.54 ± 6.55	108
Bettinger *et al*	2017	DPBR	50.9 ± 11.38	-	165
		DSBR	51.5 ± 13.71	-	129
Manrique *et al*	2019	DPBR	35.3 ± 3.8	25.3 ± 5.8	187
		DSBR	34.2 ± 4.7	26.3 ± 5.7	124
Schaeffer *et al*	2019	DPBR	50 ± 27–68	29 ± 22–49	45
		DSBR	50 (29–71)	28 (19–40)	90
Wormer *et al*	2019	DPBR	48.2 ± 11.1	26.8 ± 5.4	124
		DSBR	49.9 ± 10.7	29.5 ± 7.3	60
Suh *et al*	2020	DPBR	47.4 ± 8.6	23.7 ± 2.67	27
		DSBR	47.9 ± 8.4	22.2 ± 3.03	62
Asaad *et al*	2023	DPBR	50.4 ± 11.7	27 ± 5.2	573
		DSBR	50 ± 11.3	25.1 ± 4.4	121
Sbitany *et al*	2017	DPBR	44.8 ± 8.1	27.4 ± 5.1	84
		DSBR	48.2 ± 10.7	25.0 ± 5.2	186
Walia *et al*	2018	DPBR	51.4 ± 12.8	24.3 ± 3.6	26
		DSBR	48.6 ± 9.1	26.1 ± 5.3	109
Chandarana *et al*	2018	IPBR	51	27.3	71
		ISBR	50	25	83
Antony *et al*	2019	IPBR	47.8 ± 11.5	24.9 ± 3.3	47
		ISBR	50.2 ± 10.4	25.5 ± 4.8	57
Mirhaidari *et al*	2020	IPBR	54	27	112
		ISBR	48	26	112
Sobti *et al*	2020	IPBR	52.3 ± 11.8	28.5 ± 6.6	32
		ISBR	49.7 ± 11.0	24.8 ± 3.2	49
Manrique *et al*	2020	IPBR	54 (45–62)	25.8 (20.8–29)	55
		ISBR	47 (40–60)	24.9 (22–27.6)	69
Kim *et al*	2020	IPBR	47.68 ± 7.45	23.92 ± 3.61	53
		ISBR	46.56 ± 9.65	22.65 ± 2.81	114
Thangarajah *et al*	2019	IPBR	49.9 ± 14.8	24.7 ± 4.6	34
		ISBR	49.3 ± 11.9	24.4 ± 3.9	29
Nealon *et al*	2020	IPBR	50.7 ± 10.4	27.4 ± 5.9	183
		ISBR	50.7 ± 10.4	25.6 ± 4.4	238
Avila *et al*	2020	IPBR	46.5 ± 10.0	24.0 ± 3.9	203
		ISBR	45.9 ± 10.4	23.7 ± 4.1	202
Caputo *et al*	2021	IPBR	53	25.5	54
		ISBR	53	22	67
Patel *et al*	2021	IPBR	44.4 ± 9.1	23.4 ± 3.8	48
		ISBR	48.8 ± 12.1	23.3 ± 2.9	86
Le *et al*	2021	IPBR	51(29–69)	27.1 ± 5.3	114
		ISBR	48.5(27–73)	24.5 ± 5.2	68
Ribuffo *et al*	2021	IPBR	55.72 ± 4.5	25.36 ± 2.69	207
		ISBR	56.20 ± 7.6	24.60 ± 3.85	509
Baker *et al*	2018	IPBR	47	26 ± 4.0	43
		ISBR	48	23.4 ± 4.8	19
Bernini *et al*	2015	IPBR	47 (31–76)	23 (19–24)	39
		ISBR	51 (27–69)	23 (19–25)	34
Dikmans *et al*	2016	ISBR	43.5 ± 11.7	23.4 ± 2.9	91
		DSBR	47.3 ± 12.1	22.9 ± 2.5	92
Han *et al*	2018	ISBR	42	-	233
		DSBR	44	-	65
Azouz *et al*	2017	ISBR	-	27.6 ± 6.16	117
		DSBR	-	26.2 ± 4.81	79
Susarla *et al*	2015	ISBR	47.2 ± 10.3	24.6 ± 5.0	166
		DSBR	47.6 ± 10.2	25.4 ± 4.9	416
Viezel-Mathieu *et al*	2019	IPBR	46.5 ± 11.3	-	60
		DSBR	50.9 ± 11.1	-	56
Finkelstein *et al*	2023	IPBR	52.7 ± 11.1	27.6 ± 5.6	201
		DPBR	50.8 ± 10.1	26.4 ± 5.5	147
Zingaretti *et al*	2024	IPBR	49.47	22.71	51
		DPBR	52.38	24.05	56

DPBR, delayed prepectoral breast reconstruction; DSBR, delayed subpectoral breast reconstruction; IPBR, immediate prepectoral breast reconstruction; ISBR, immediate subpectoral breast reconstruction.

### Overall complications

**Figure 2.**
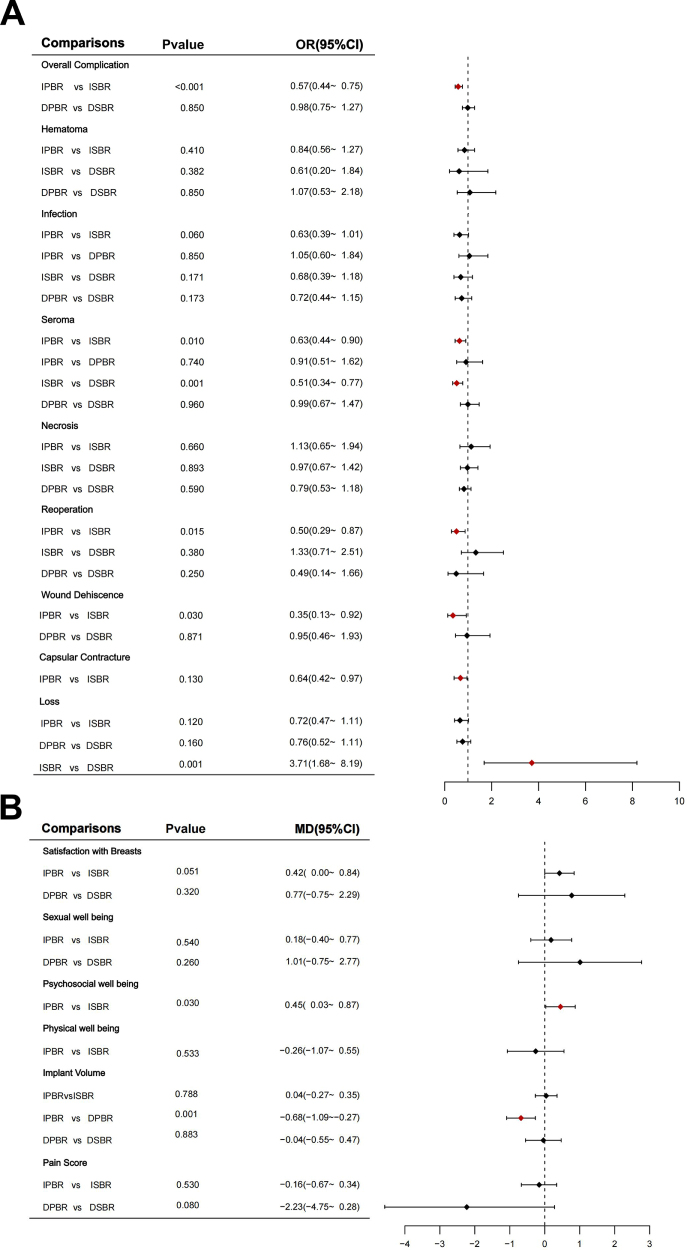
Forest plot comparison of the different surgical methods for all outcomes. OR, odds ratio; MD, mean difference; DPBR, delayed prepectoral breast reconstruction; DSBR, delayed subpectoral breast reconstruction; IPBR, immediate prepectoral breast reconstruction; ISBR, immediate subpectoral breast reconstruction.

**Figure 3.**
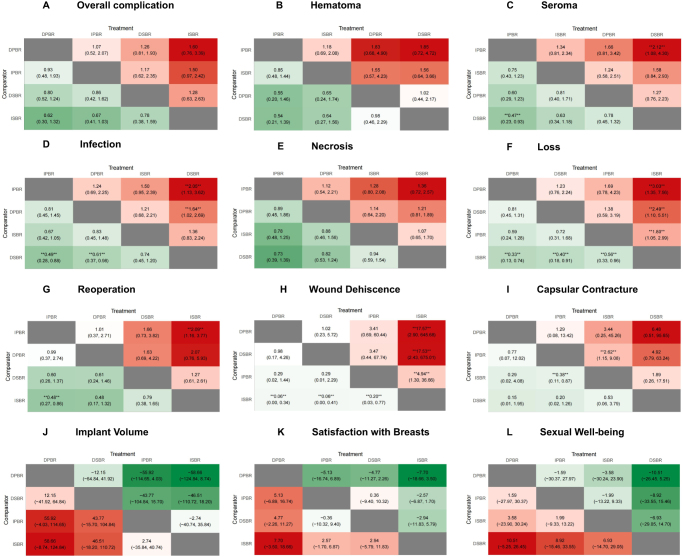
League table heat plots for the four treatment strategies. A position closer to the top left indicates a more favorable outcome. Statistically significant differences are marked with **. DPBR, delayed prepectoral breast reconstruction; DSBR, delayed subpectoral breast reconstruction; IPBR, immediate prepectoral breast reconstruction; ISBR, immediate subpectoral breast reconstruction.

**Figure 4.**
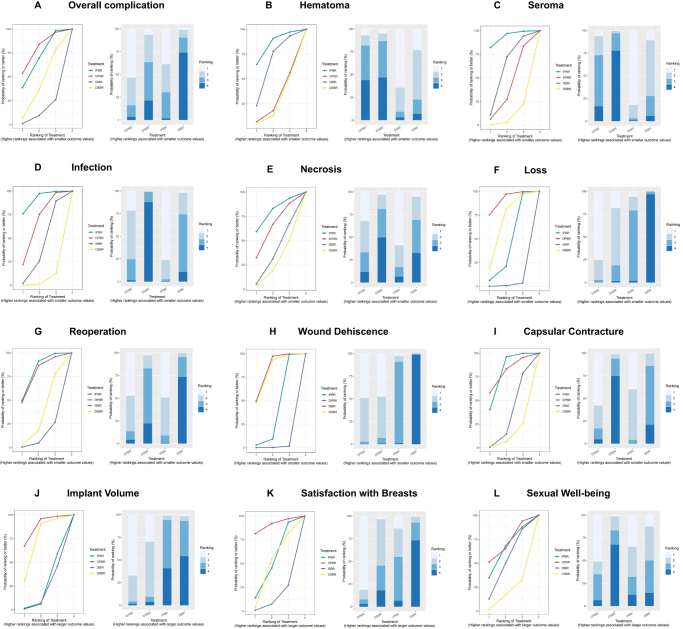
The cumulative ranking curves and rank probability plots for the four treatment strategies. Cumulative ranking curves (left): A larger area under the curve corresponds to a higher SUCRA value, indicating a better method. Rank probability plots (right): the areas of each rank illustrate the probability of each treatment being ranked first, second, third, or fourth. DPBR, delayed prepectoral breast reconstruction; DSBR, delayed subpectoral breast reconstruction; IPBR, immediate prepectoral breast reconstruction; ISBR, immediate subpectoral breast reconstruction.

According to the PMA, IPBR was associated with fewer overall complications than ISBR (Fig. 2A). In contrast, the NMA did not detect statistically significant differences among the four strategies (Fig. 3A). According to the SUCRA, DPBR ranked highest, followed by IPBR; ISBR ranked lowest (Fig. 4A). This ordering is consistent with the results of the PMA, which revealed fewer overall complications with IPBR than with ISBR.

### Hematoma

No statistically significant differences were observed in the incidence of hematoma across the four surgical methods according to either the PMA or the NMA (Fig. 2A, Figure 3B, Figure 4B). The SUCRA rankings provided additional insight and indicated that the IPBR (0.85) and ISBR (0.65) approaches may be more favorable than the DPBR and DSBR approaches in terms of lower bleeding risk (Table 2).

**Table 2:** The surface under the cumulative ranking curve values for all the outcomes.

Groups	Overall complications	Hematoma	Seroma	Infection	Necrosis	Loss	Reoperation	Wound dehiscence	Capsular contracture	Implant volume	Satisfaction with breasts	Sexual well-being
DPBR	0.78	0.27	0.39	0.65	0.63	0.91	0.77	0.82	0.79	0.87	0.90	0.70
DSBR	0.41	0.24	0.09	0.05	0.24	0.66	0.33	0.80	0.11	0.72	0.47	0.17
IPBR	0.69	0.84	0.93	0.91	0.78	0.42	0.82	0.37	0.79	0.22	0.51	0.64
ISBR	0.12	0.65	0.59	0.39	0.35	0.01	0.11	0.01	0.31	0.18	0.12	0.49

DPBR, delayed prepectoral breast reconstruction; DSBR, delayed subpectoral breast reconstruction; IPBR, immediate prepectoral breast reconstruction; ISBR, immediate subpectoral breast reconstruction.

### Seroma

Direct evidence supported a seroma gradient (IPBR < ISBR < DSBR). After indirect comparisons were integrated, the NMA retained the IPBR < DSBR contrast, while the SUCRA ranked IPBR first (Fig. 3C). More results involving the SUCRA values for seroma are shown in Table 2 and Figure 4C.

### Infection

The PMA did not detect statistically significant differences among the four strategies (Fig. 2A). However, the NMA revealed fewer postoperative infections with IPBR and DPBR than with DSBR (Fig. 3D). The SUCRA supported this ordering, placing IPBR first (0.91) and DPBR second (0.65), with DSBR very low (0.05), a finding that is consistent with the highest infection risk (Table 2, Figure 4D).

### Necrosis

Across the PMA and NMA, no comparison reached statistical significance, which indicates a broadly similar risk of flap necrosis across the four strategies (Fig. 2A; Figure 3E). Nevertheless, the SUCRA suggested a gradient that favors prepectoral strategies (Fig. 4E). Compared with subpectoral approaches, IPBR and DPBR were associated with higher probabilities of lower flap necrosis risk (0.79; 0.62; Table 2).

### Loss

In the PMA, compared with ISBR, DSBR had a lower risk of implant loss; the other pairwise contrasts were not significant. NMA confirmed a lower risk of loss with DPBR, DSBR, and IPBR than with ISBR, but no meaningful differences were detected among the DPBR, DSBR and IPBR strategies. The SUCRA rankings are shown in Figure 4F and Table 2: DPBR 0.91, DSBR 0.66, IPBR 0.42, and ISBR 0.01.

### Reoperation

After indirect evidence was integrated, the risk of reoperation was lower for IPBR than for ISBR, and the risk was estimated to be lower for IPBR than for ISBR (OR: 0.48; 95% CI: 0.27–0.86; Figure 3G). As shown in Figure 4G, the SUCRA supported this ordering and ranked IPBR 0.80 and DPBR 0.77 the highest, this suggests the superior performance of prepectoral strategies for this outcome.

### Wound dehiscence

Notable differences in wound dehiscence were observed across the four strategies. Both the PMA and the NMA indicated that compared with ISBR, IPBR was associated with a significantly lower risk of this complication (OR: 0.20; 95% CI: 0.03–0.77). NMA further revealed that compared with ISBR, DPBR, DSBR, and IPBR were associated with significantly lower risks (Fig. 3H, Figure 4H). In terms of the SUCRA probabilities (Table 2), DPBR (0.82) ranked highest, followed by DSBR (0.80), IPBR (0.37), and ISBR (0.01).

### Capsular contracture

According to the PMA, compared with ISBR, IPBR had a lower rate of capsular contracture. The NMA also indicated lower contracture with IPBR than with ISBR (OR: 0.38, 95% CI 0.11–0.97; Figure 3I, Figure 4I). Consistent with those results, the SUCRA placed DPBR (0.78) and IPBR (0.79) toward the favorable end of the distribution, with ISBR lower at 0.31.

### Implant volume

According to the PMA, the implant volume was greater in the DPBR group than in the IPBR group, whereas the difference between the DPBR and DSBR groups and that between the IPBR and ISBR groups were not significant (Fig. 2B). The NMA did not detect overall differences among the four strategies. As shown in Table 2 and Figure 4J, the rank probabilities favored delayed approaches: the SUCRA ranked DPBR first (0.87), followed by DSBR (0.72), IPBR (0.22), and ISBR (0.18).

### BREAST-Q

Across the BREAST-Q, only the “satisfaction with breasts” and “sexual well-being” scales could be included in the NMA. Neither the PMA nor the NMA detected significant differences among the four strategies in terms of either outcome. With respect to breast satisfaction, the SUCRA clearly ranked DPBR higher than the other strategies (0.90), as IPBR and DSBR were intermediate, and ISBR was the lowest ranked strategy. In the sexual well-being analyses, the SUCRA probabilities similarly placed DPBR first (Table 2). Overall, the evidence consistently highlights DPBR as the top performer within the BREAST-Q (Fig. 4K, Figure 4L).

Some evidence is limited to direct comparisons. In the PMA, IPBR was associated with higher psychological well-being than was ISBR, whereas physical well-being did not differ significantly between IPBR and ISBR (Fig. 2).

### Pain score

With respect to pairwise evidence, the meta-analysis did not identify meaningful differences in pain between IPBR and ISBR or between DPBR and DSBR (Fig. 2).

## Discussion

This is the first NMA that simultaneously evaluates four widely used BR strategies (IPBR, ISBR, DPBR, and DSBR) for surgical safety and patient experience. Earlier reviews relied on PMAs, which may not fully account for the differences. By carefully examining each original study, we aimed to minimize the potential influence of interstudy heterogeneity on our conclusions. SUCRA values were calculated to quantify the relative ranking of strategies for each outcome.

The PMA indicated a lower overall complication rate with IPBR than with ISBR, whereas the NMA revealed no statistically significant differences across the four strategies. Previous studies by Li *et al*^[^53^]^ and Ostapenko *et al*^[^54^]^ revealed no difference between prepectoral and subpectoral planes, but they did not stratify the planes by timing. According to the SUCRA, prepectoral placement tended to perform better overall, which potentially reflects lower rates of surgical trauma when muscle dissection is avoided. Delayed strategies ranked higher within the same plane, which is consistent with the lower flap tension and lower rate of ischemic complications associated with TE use. The observation that IPBR was ranked ahead of DSBR may indicate that the plane exerts a stronger effect on overall complications than timing does.

Seroma is a common early complication after reconstruction. It is plausible that without stratification by timing, no difference by plane was observed in prior work^[^55,56^]^. In our study, compared with ISBR, IPBR appeared to have a lower seroma rate. Subpectoral approaches require muscle dissection, which may increase potential spaces and exudative surfaces. Conversely, prepectoral reconstruction limits dissection and reduces dead space^[^57^]^. In delayed reconstruction, insufficient TE filling may maintain dead space and promote seroma formation^[^58^]^. Additional TE filling has been reported in recurrent seroma^[^59^]^. The SUCRA further suggested that the influence of timing is stronger than that of plane. Interestingly, the hematoma ranking paralleled that of seroma, possibly because of muscle dissection and larger potential spaces. No significant differences were detected across strategies, a result that is likely due to effective intraoperative controls.

Infection after BR is a critical indicator of surgical safety. Our PMA generally revealed no significant differences. In contrast, the NMA suggested a lower infection risk for IPBR and DPBR than for DSBR. A plausible explanation for this result is that prepectoral reconstruction reduces surgical trauma and potentially limits drain time. However, many prior studies did not detect significant differences, which suggest that infection risk may be influenced by multiple contributing factors^[^53,56^]^. Thus, plane and timing alone should not guide decisions, and rigorous perioperative management and infection prevention should be prioritized.

Flap necrosis is among the most serious early complications after reconstruction. In our analysis, no significant differences were detected among the four strategies. The SUCRA suggested a better ranking of prepectoral approaches. Abbate *et al* also reported lower flap necrosis in prepectoral cases^[^60^]^. This pattern may be related to case selection because surgeons often use intraoperative perfusion assessment to reduce necrosis in prepectoral groups^[^61^]^.

Wound dehiscence and implant loss are key markers of reconstructive failure. We found lower rates of both outcomes with DPBR, DSBR, and IPBR than with ISBR. Subpectoral reconstruction requires pectoralis elevation and pocket creation. Dynamic muscle forces may further increase tension, whereas muscle division may impair flap perfusion, which together provide a plausible pathway to wound dehiscence^[^62,63^]^. Compared with immediate reconstruction, delayed reconstruction results in less dehiscence, which likely reflects tissue expansion that slowly enlarges the pocket and reduces tension. Consistently, the SUCRA suggested that timing may have a greater influence on dehiscence than plane does. Loss refers to the removal of the implant due to severe complications. ISBR appeared to be associated with the greatest risk, which is consistent with its higher overall complication rate. The SUCRA suggested a benefit of delayed over immediate reconstruction, a finding that supports the potential role of flap tension in driving the development of severe events.

Capsular contracture is a late, multifactorial complication that is likely related to infection, adjuvant therapy, and a scarring phenotype. We observed lower contracture with IPBR than with ISBR, a result that is consistent with the findings of Li *et al*^[^53^]^. Although the NMA did not detect significant differences across strategies, the SUCRA favored the prepectoral approach over the subpectoral approach. Sobit *et al*^[^34^]^ reported a role for pectoralis fibrosis and contraction. Prepectoral approaches avoid muscle intervention and may reduce this risk^[^58^]^. As adjuvant radiotherapy has been linked to increased capsular contracture, delayed reconstruction may help manage the timing of implantation around radiotherapy^[^64,65^]^. Overall, we detected no significant differences, which suggests that the contracture rates are likely comparable among techniques when radiotherapy is not administered. In patients for whom radiation is expected, strategies should be carefully selected, and prepectoral reconstruction should be considered.

Reoperation was evaluated as an unplanned surgical return for the management of complications or defects. In our study, IPBR appeared to have a lower reoperation rate than ISBR, a finding that is consistent with the pattern observed for capsular contracture. Earlier reports identified contracture as one of the principal causes of reoperation^[^66^]^. The SUCRA rankings indicated a greater disadvantage with subpectoral approaches. Owing to the higher complication rate seen in our data, ISBR may be associated with a higher likelihood of additional procedures. In contrast, the timing effect was not clear according to the SUCRA and thus needs to be investigated further.

Implant volume influences both the risk of complications and patient satisfaction. Our results reveal larger volumes with delayed versus immediate reconstruction. The SUCRA analysis indicated that delayed reconstruction ranked higher than immediate reconstruction. Furthermore, the results suggested that the prepectoral approach was superior to the subpectoral approach. Those findings may reflect the volume gains from staged TE use in delayed reconstruction. Immediate procedures are often limited by flap tension and skin elasticity; thus, very large fills are usually avoided in direct-to-implant cases^[^67^]^. Several studies have also suggested that prepectoral reconstruction, with support meshes and a refined pocket design, can lead to the use of more fill without increasing complication rates. Subpectoral reconstruction may be limited by the pectoralis muscle^[^68,69^]^. From a practical standpoint, immediate reconstruction is more suitable for conservative breast sizing. However, delayed reconstruction is preferable for larger-volume targets and higher esthetic expectations, including contralateral adjustments.

BR can improve quality of life and increase patient satisfaction after mastectomy. The BREAST-Q instrument is used to assess subjective satisfaction^[^70^]^. According to the BREAST-Q results, the overall appearance scores are largely similar among the techniques^[^71^]^. The SUCRA suggested better rankings for prepectoral versus subpectoral approaches with respect to breast satisfaction and sexual well-being, results that may be due to smaller operative fields, lower complication rates, and greater volume potential. Delayed reconstruction can optimize esthetics, which increases patient satisfaction. However, the impact is variable. Some studies indicate benefits with immediate reconstruction, especially in younger women seeking rapid restoration of body image and confidence in relationships. Other studies favor delayed reconstruction for a larger volume and higher esthetic expectations^[^15^]^. Surgical choices should be personalized to patient preferences and clinical factors.

The PMA reported psychological well-being, physical well-being, and pain. Psychosocial well-being refers to the subjective psychological and social feelings of patients after reconstruction^[^72^]^. In our analysis, the prepectoral approach was superior to the subpectoral approach. Possible explanations include chronic chest tightness and restricted motion after subpectoral placement, which may decrease patient satisfaction. Conversely, prepectoral reconstruction is often described as more comfortable with fewer activity limitations, which enables patients to “forget” about implant and achieve better psychosocial outcomes^[^73,74^]^.

For pain, we observed no significant difference between planes. Mégevand *et al*^[^75^]^ and Ching *et al*^[^55^]^ reported a lower frequency of pain with the prepectoral approach because muscle dissection is avoided. Copeland-Halperin *et al*^[^76^]^ reported a 33% reduction in days of opioid use with prepectoral expanders, and Kim *et al*^[^35^]^ reported reduced inpatient opioid use with prepectoral expanders. Other investigators reported no difference^[^77^]^. Variation in analgesic standards likely contributes to any observed differences. Larger randomized trials with uniform pain standards would help clarify the true effect.

Economic costs are a critical factor in selecting the optimal reconstruction strategy. From an economic perspective, immediate reconstruction generally demonstrates superior direct cost-effectiveness when the number of surgical stages is reduced. Within delayed reconstruction, the choice of plane also impacts costs. Unlike subpectoral reconstruction, prepectoral reconstruction does not involve muscle resistance, thus allowing faster and more comfortable expansion and requiring fewer clinical visits^[^27,68^]^; it not only lowers direct medical costs but also reduces the indirect costs associated with medical commuting. Beyond economic factors, patient preferences ultimately dictate the final decision. For patients prioritizing a rapid return to reduce both trauma and costs, immediate reconstruction is the preferred choice. In contrast, delayed reconstruction, especially the prepectoral approach, may be better favored by patients who have high expectations with regard to breast shape and volume. According to our SUCRA rankings, patients who have adequate tissue perfusion and prioritize safety may lean toward IPBR. However, for patients with high-risk flaps, the subpectoral plane may be the preferred choice; the muscle can provide essential protection.

This study has several limitations. The data are retrospective, which introduces confounding factors that cannot be fully eliminated. Clinical factors such as planned radiotherapy, smoking status, and body mass index were not consistently available. Important technical factors, including mesh use, TE protocols, drain policies, and perioperative antibiotics, varied across studies. The definitions and timing of outcomes, including pain scale scores, BREAST-Q scores, and follow-up duration, were also not uniform. Therefore, we were unable to analyze the specific impact of these factors on surgical outcomes. Finally, some late outcomes are underrepresented. These constraints may attenuate or inflate between-group differences.

## Conclusions

Both plane and timing can influence complications and patient experience. Subpectoral reconstruction, which includes muscle dissection, may increase complication rates but has fewer constraints on flap quality. Prepectoral reconstruction avoids muscle elevation and may reduce complications but typically requires favorable flaps and careful evaluation before and during surgery. Immediate reconstruction can shorten recovery, lower costs, and support earlier psychological gains but provides a limited opportunity to adjust volume. Delayed reconstruction allows volume optimization, esthetic refinement, and flexible coordination with radiotherapy, although it increases follow-up demands and financial burden. A comprehensive surgical plan should be based on the benefits and limitations of flap quality, recovery burden, and patient goals to individualize treatment selection.

## Data Availability

All data used in the study were extracted from published articles and their supplementary files.

## Research registration unique identifying number (UIN)

This network meta-analysis was registered in the PROSPERO international database (NO. CRD420251034280, https://www.crd.york.ac.uk/prospero/).
